# Acute hydrocephalus caused by colloid cyst of third ventricle: A case report

**DOI:** 10.1016/j.radcr.2023.07.037

**Published:** 2023-08-05

**Authors:** Prajwal Dahal, Sharma Paudel, Ongden Yongen Tamang, Rudra Prasad Upadhyay, Sabina Parajuli, Kiran Kayastha, Prakash Kayastha

**Affiliations:** a Department of Radiology and Imaging, Grande International Hospital, Kathmandu, Nepal; b Department of Radiology and Imaging, Tribhuvan University Teaching Hospital, Kathmandu, Nepal; c Department of Pathology, Bir Hospital, Kathmandu, Nepal; d Emergency Department, Grande International Hospital, Kathmandu, Nepal

**Keywords:** Colloid cyst, Hydrocephalus, Third ventricle

## Abstract

Colloid cysts (CCs) of third ventricle are rare benign lesions. They present with acute hydrocephalus and its sequalae like brain herniation, infarcts resulting even death in otherwise healthy individual. We present a case of an acute hydrocephalus caused by CC of third ventricle. A middle age male was airlifted from a remote district of Nepal to our hospital with no accompanying doctor. The patient had headache, multiple episodes of vomiting, abnormal body movement, and loss of consciousness for 24 hours. On examination, vitals were stable; the Glasgow Coma Scale (GCS) score was 7. The patient was intubated in emergency and an MRI brain was done. MRI showed an obstructive lesion in third ventricle with features consistent with CC and an active hydrocephalus. There were multifocal infarcts in the bilateral cerebrum, left part of mid brain and pons, left thalamus and left superior cerebellum. We inserted external ventricular drainage in emergency operation theatre within hours and endoscopic excision of the lesion was done on the next day. In histopathology, the lesion was confirmed to be a CC.

## Introduction

Colloid cysts (CCs) account for 1% of all intracranial tumors [1]. In noncontrast CT, CCs are typically unilocular and hyperdense with no calcification. In MRI, the lesion is typically hyperintense in T1-weighted images [1]. The signal in T2-weighted images is variable [1,2]. Small CCs remain asymptomatic. However, if they obstruct Cerebrospinal fluid (CSF) flow, acute complications are seen [2,3]. Management of CCs is challenging due to their deep location. Surgical resection is the mainstay of treatment.

## Clinical presentation

A 35-year-old male was airlifted with no accompanying doctor from the Bhojpur district of Nepal. According to the patient's sister, he complained of severe headache, multiple episodes of vomiting for 24 hours. He also had multiple episodes of abnormal body movement, and loss of consciousness in the past 24 hours. The patient also complained of dizziness for 1 month which he attributed to typhoid fever he had 1 month back. The typhoid fever was diagnosed and treated in Bhojpur. He was a known hypertensive under regular medication for 2 years. On examination, vitals were stable, and a low Glasgow Coma Scale (GCS) score of 7 was seen.

## Imaging

**Fig. 1:**
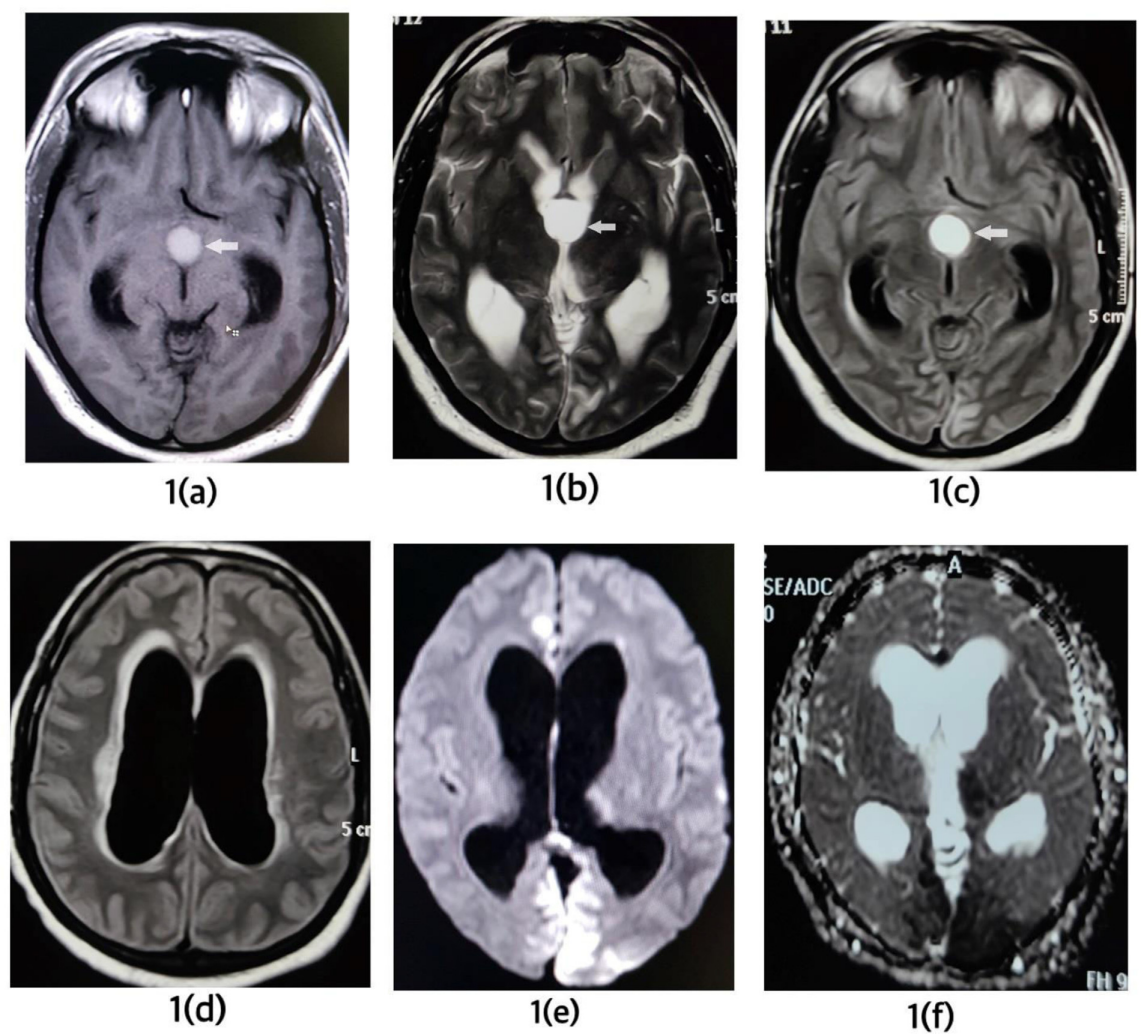
(A and B) are T1- and T2-weighted MRI images showing high signal intensity cyst (pointed by an arrow) in third ventricle. (C) is FLAIR image showing high signal of colloid cyst. (D) is fluid attenuated inversion recovery (FLAIR) image showing active hydrocephalus with mild periventricular ooze. (E) is diffusion-weighted image and (F) is ADC image showing infarcts in bilateral occipital lobes and left thalamus. There were infarcts in left part of midbrain, pons, left thalamus, and left superior cerebellum. CC did not show diffusion restriction.

In the emergency department, the patient was immediately intubated and an MRI of the brain was done (Fig. 1).

**Fig. 2:**
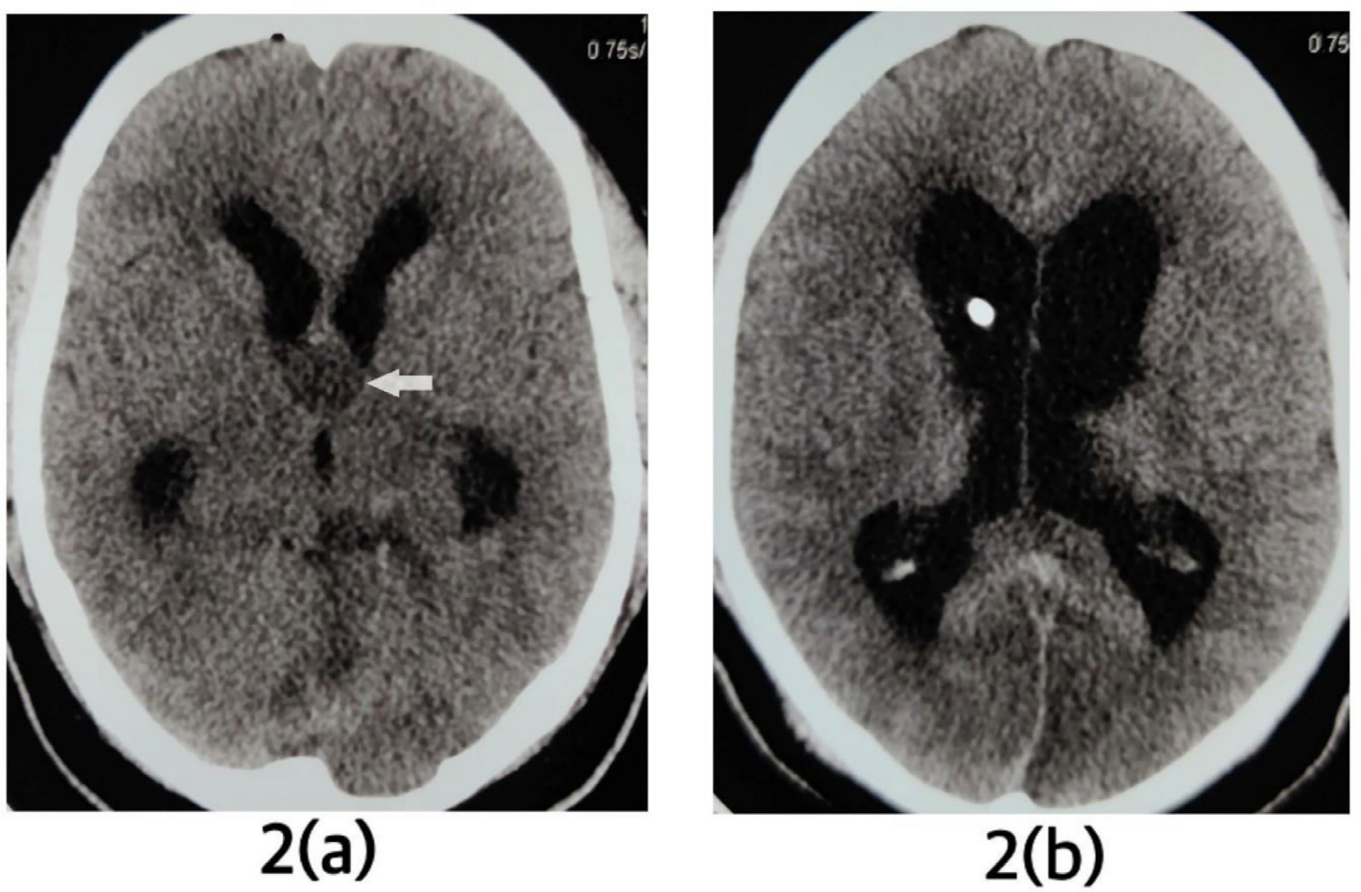
(A) shows hypodense colloid cyst in third ventricle (pointed by an arrow). In (B), an external ventricular drainage is seen in right lateral ventricle. Mild decompression of ventricular system is seen. Periventricular ooze has mildly reduced.

A well-defined cystic lesion measuring ∼1.8 × 1.8 cm was seen in the anterosuperior aspect of the third ventricle. The lesion showed high signal in both T1- and T2-weighted images. The high signal in T2 was not suppressed inFLAIR. Diagnosis of CC was made. There was active noncommunicating hydrocephalus with mild periventricular ooze. Diffusion restriction was seen in bilateral occipital lobes (posterior cerebral artery territories), part of the midbrain, pons, left thalamus, and left superior cerebellum (left superior cerebellar artery territory). After immediate intubation in emergency, preoperative blood investigations were done. The patient was shifted to emergency OT. External ventricular drainage was kept in the right Kocher's point. A plain CT scan of head was done after placement of external ventricular drainage. In plain CT, the CC appeared hypodense and because of the drainage, the ventricular system was mildly decompressed (Fig. 2).

Further clinical deterioration was not seen after the drainage. The patient was admitted in ICU.

## Differential diagnosis

With the clinical history and imaging findings in our case, no differential diagnosis needs to be considered. Imaging wise, the lesion is a typical colloid cyst. However, in case of atypical lesions in imaging, hyperdense meningioma, giant cell astrocytoma and intraventricular neurocysticercosis can be the differential diagnosis.

## Case management and treatment

**Fig. 3:**
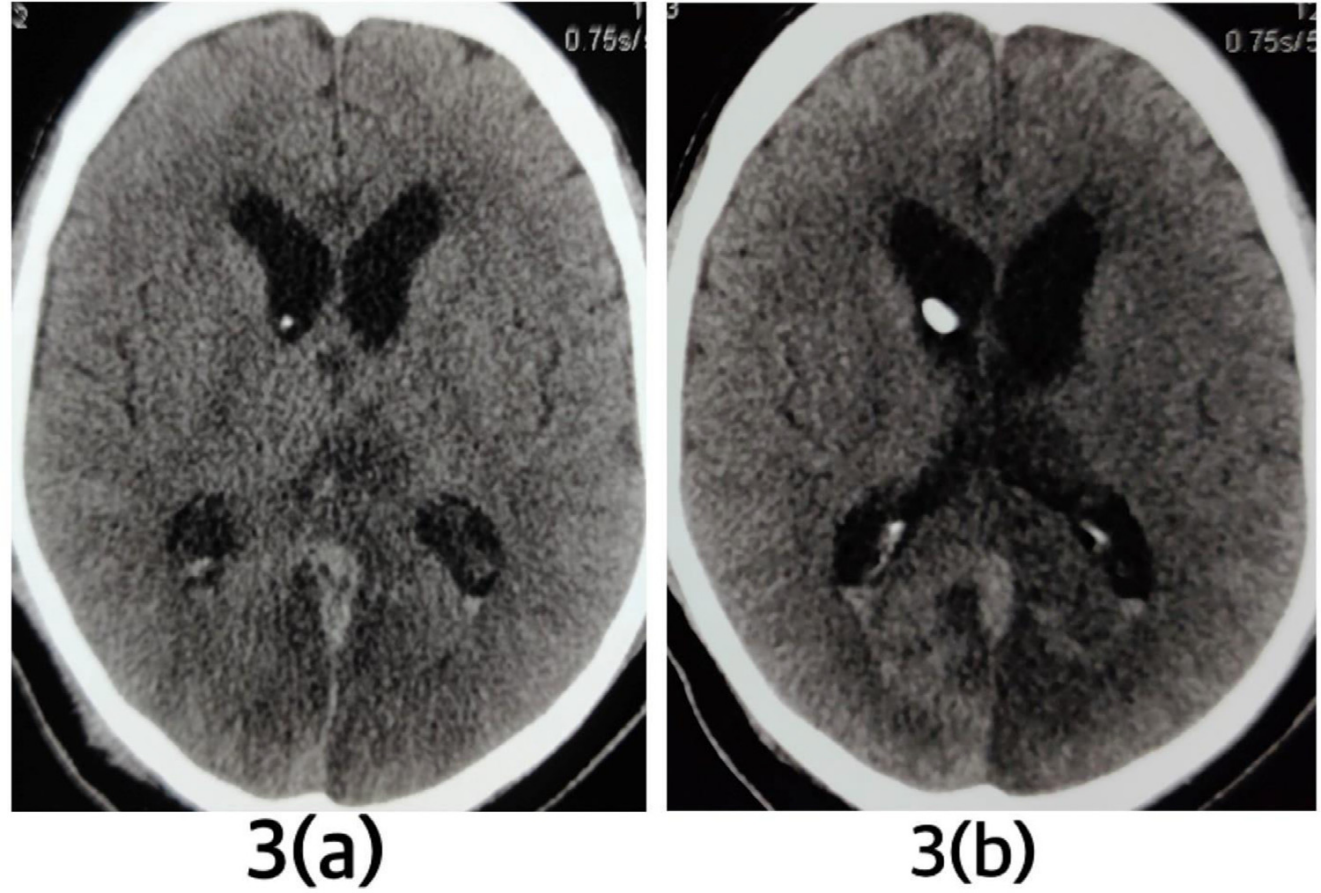
Plain CT of head after endoscopic resection of CC and septal fenestration. (A) There is absence of colloid cyst in third ventricle. (B) An external ventricular drainage is seen in situ and the ventricular system is decompressed.

The next day, resection of the colloid cyst with septal fenestration was done by endoscopic intraventricular approach (from frontal horn). Plain CT scan of head was repeated after the surgery. CC was absent in third ventricle and the ventricular system was further decompressed (Fig. 3).

**Fig. 4:**
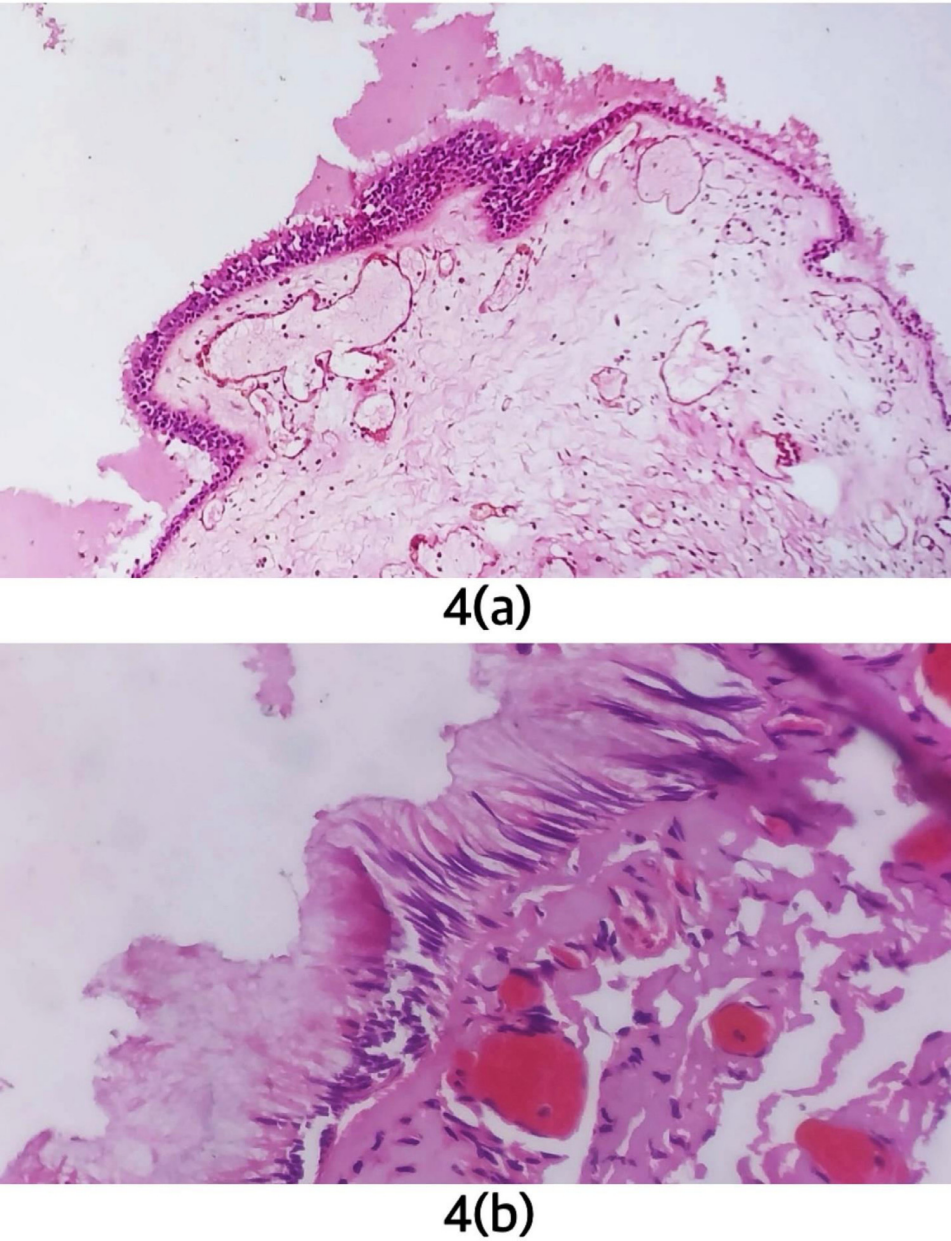
Histological images of resected specimen. (A) is low power view. The image shows a cystic lesion with fibrous wall and eosinophilic contents within. (B) is high power view showing cuboidal, columnar, and pseudostratified columnar cells lining the wall of CC.

In histology (Fig. 4), a cyst with fibrous wall was seen. The fibrous wall was lined by ciliated columnar epithelium and pseudostratified epithelium. The cyst contained eosinophilic material with a few mixed inflammatory cells consisting of lymphocytes, plasma cells, eosinophils, and neutrophils. The lesion was confirmed to be a third ventricular CC.

**Fig. 5:**
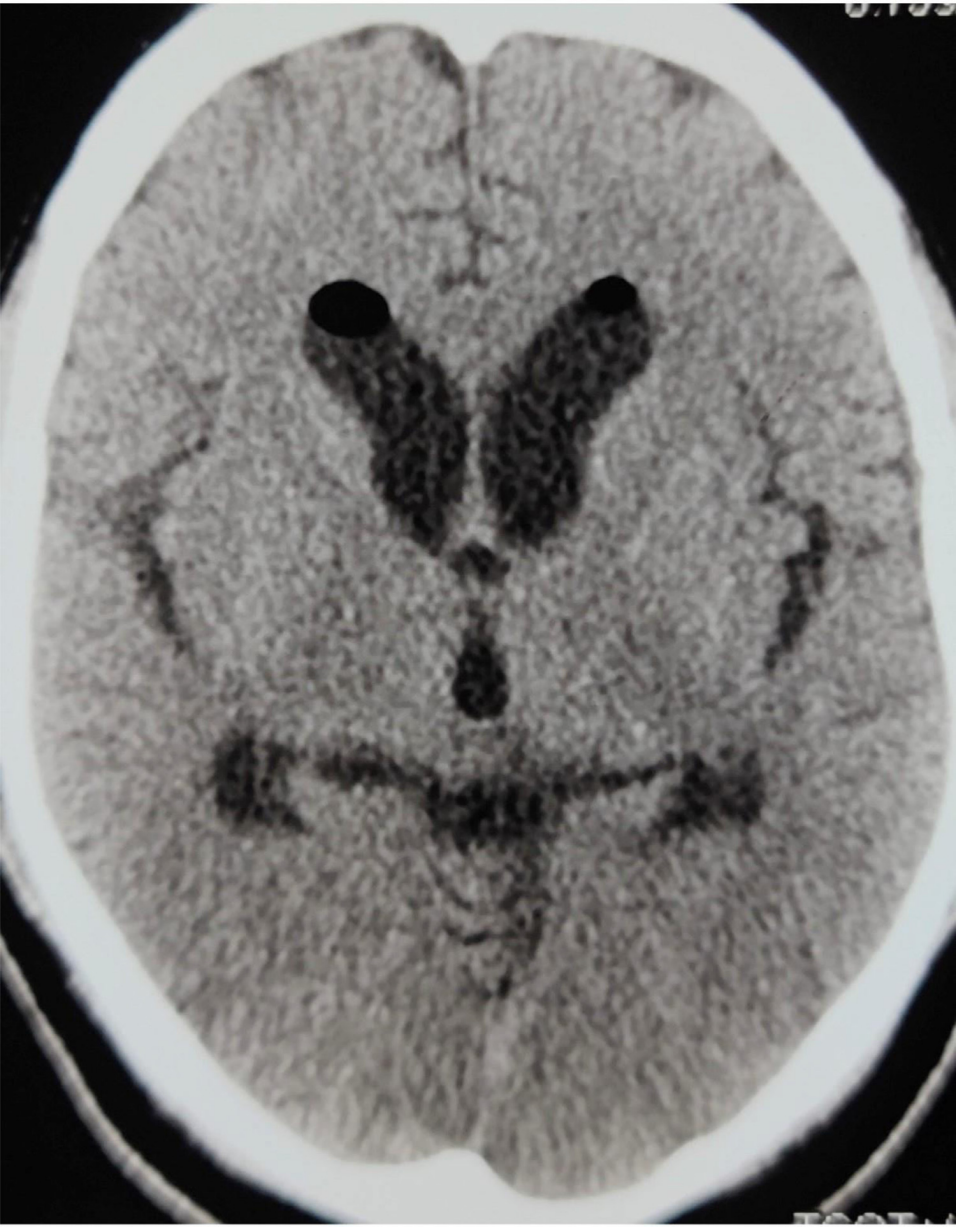
Plain CT head after removal of external ventricular drainage after 10 days. Hydrocephalus has completely resolved. Mild air is present in frontal horns of bilateral lateral ventricles.

The patient was admitted to ICU. After surgery, the patient showed gradual clinical improvement and was extubated. Since permanent shunting was not required, external ventricular drainage was removed after 10 days. Plain CT of head done after removal of external ventricular drainage. Fig. 5 shows complete resolution of hydrocephalus.

At the time of discharge, GCS of the patient was 14. The patient was ambulatory with minimal support and feeding orally. Talks were occasionally irrelevant. Bilateral pupils were round, reactive to light. Ptosis was present in right eye and exotropia in left eye for which eye muscle exercise were taught. Regular physiotherapy was advised at the time of discharge.

## Outcome and follow-up

The patient visited neurosurgery OPD after 3 weeks. GCS was 14 with irrelevant talks at times. Mild weakness was present in all 4 limbs. Ptosis and exotropia were still present. The patient came for few follow-up visits and was alive for 1 year from the incident. The patient succumbed to COVID-19 infection after a year. No autopsy was performed.

## Discussion

CCs of the third ventricle are the most common neuroepithelial cysts [1]. They are congenital lesions of endodermal origin [3,4]. CCs are seen in approximately 3 individuals per million per year [1,2]. Smaller cysts are asymptomatic; larger cysts sometimes result in acute noncommunicating hydrocephalus, brain herniation, and feature of raised intracranial pressure like papilledema, headache, vomiting, seizure, etc. [5]. Colloid cysts are usually present in the third ventricle but may be seen in other ventricles and sometimes outside the ventricular system [1]. CCs attached to the roof of the third ventricle may be pendulous and cause intermittent ventricular obstruction [1,2]. Diagnosis is usually suspected in imaging and confirmed in histopathology. CCs rich in protein and cholesterol are hyperdense in CT and show a high signal in T1 images and a low signal in T2 images. Contents of these kinds of cysts are viscous and aspiration cannot be done [1,6]. Fifty percent of CCs show high signals in T1-weighted images [1,3]. Signals in T2 and FLAIR are more variable [3]. Homogeneous high signal may be seen or the central low signal and peripheral rim of the high signal can be seen or the high signal with a low signal central dot (size of dot being less than 5 mm) can be seen in T2 and FLAIR images [2,3,6]. Colloid cysts with low protein and cholesterol contents show fluid signals in all MRI sequences [3]. Subtle discontinuous peripheral rim enhancement may be seen in some of the lesions which is likely due to enhancement of adjacent vessels rather than true enhancement [3]. Diffusion restriction and susceptibility changes in GRE/SWI images are not seen. In a study done by Khanpara et al. [3], homogenous T2 hyperintense cysts and cysts with dot signs were associated with a higher risk of obstructive ventriculomegaly [7]. T2 hyperintense cysts are more likely to be symptomatic compared to T2 hypointense cysts. A widely accepted theory is that T2 hyperintensity is associated with a high concentration of cholesterol. There is an osmotic influx of fluid and the size of the cyst increases and it becomes symptomatic [7]. In our case also, the cyst was T2 hyperintense. Despite variation in imaging characteristic of CCs, the lesions are similar in histology. CCs have thin fibrous wall lined by single to pseudostratified cuboidal or columnar epithelium and occasional goblet cells [1], [2], [3]. The contents are acellular and gelatinous. Management of colloid cysts is influenced by age and performance status of the patient, the size of CC, and the presence of hydrocephalus. Small (<10 mm size) nonobstructive CCs can be followed up [3,4]. We should manage complications of acute hydrocephalus too. Multifocal infarcts developed in our case because of compression of cerebral arteries due to hydrocephalus. External ventricular drainage was kept within hours to prevent further hydrocephalus and extension of infarct. Management options for large and symptomatic CCs include stereotactic aspiration, endoscopic fenestration, and resection, open surgical resection, and microsurgery [4,6,8]

## Patient consent

All the images used in this manuscript are anonymized.

Consent for the publication of this case report was obtained from the patient.
